# Microarray based analysis of an inherited terminal 3p26.3 deletion, containing only the *CHL1 *gene, from a normal father to his two affected children

**DOI:** 10.1186/1750-1172-6-12

**Published:** 2011-04-01

**Authors:** Cristina Cuoco, Patrizia Ronchetto, Stefania Gimelli, Frédérique Béna, Maria Teresa Divizia, Margherita Lerone, Marisol Mirabelli-Badenier, Monica Mascaretti, Giorgio Gimelli

**Affiliations:** 1 Laboratorio di Citogenetica, Istituto G. Gaslini, 16147 Genova, Italy; 2 Service of Genetic Medicine, University Hospitals of Geneva, 1211 Geneva, Switzerland; 3 Servizio di Genetica Molecolare, Istituto G. Gaslini, 16147 Genova, Italy; 4 Divisione di Neuropsichiatria Infantile, Istituto G. Gaslini, 16147 Genova, Italy

## Abstract

**Background:**

terminal deletions of the distal portion of the short arm of chromosome 3 cause a rare contiguous gene disorder characterized by growth retardation, developmental delay, mental retardation, dysmorphisms, microcephaly and ptosis. The phenotype of individuals with deletions varies from normal to severe. It was suggested that a 1,5 Mb minimal terminal deletion including the two genes *CRBN *and *CNTN4 *is sufficient to cause the syndrome.

In addition the *CHL1 *gene, mapping at 3p26.3 distally to *CRBN *and *CNTN4*, was proposed as candidate gene for a non specific mental retardation because of its high level of expression in the brain.

**Methods and Results:**

we describe two affected siblings in which array-CGH analysis disclosed an identical discontinuous terminal 3p26.3 deletion spanning less than 1 Mb. The deletion was transmitted from their normal father and included only the *CHL1 *gene. The two brothers present microcephaly, light mental retardation, learning and language difficulties but not the typical phenotype manifestations described in 3p- syndrome.

**Conclusion:**

a terminal 3p26.3 deletion including only the *CHL1 *gene is a very rare finding previously reported only in one family. The phenotype of the affected individuals in the two families is very similar and the deletion has been inherited from an apparently normal parent. As already described for others recurrent syndromes with variable phenotype, these findings are challenging in genetic counselling because of an evident variable penetrance.

## Introduction

The 3p deletion syndrome is a rare contiguous gene syndrome caused by deletions in the 3p25-pter region. The deletions are variable in size, ranging from one to several megabases, they don't present common breakpoints and mostly occur *de novo*, but a few familial cases have been reported [1-4]. The syndrome is characterized by a recognizable phenotype including low birth weight, growth and mental retardation, developmental delay and characteristic facial appearances. The clinical manifestations in individuals with 3p deletions vary from normal to severe. A milder phenotypic effect or a normal intelligence [4,5] has also been described for larger [3,6], often inherited, deletions of this region [1-4,7,8] and appears to be secondary to the breakpoint's location and the deletion extent [1-3]. Moreover, cases with minimal pathological features despite the presence of a large terminal 3p deletion have been described [3,4,9].

Recently, a cohort of 14 patients with visible distal 3p deletions has been studied by SNP array to better define the genetic basis of 3p deletion syndrome [10]. Among the different haploinsufficient genes, *CRBN *and *CNTN4 *have been indicated as sufficient to cause the typical clinical features [9] while the *CHL1 *gene has been suggested to contribute to mental development [4,8,11].

We describe a sub microscopic 3p26.3 terminal deletion transmitted from the normal father to his two affected children. The imbalance is less than 1 Mb in size and includes only the gene *CHL1*, a member of the L1 family of cell adhesion molecules previously suggested to be responsible for mental defects in patients with 3p- syndrome.

## Methods

Karyotyping was performed on peripheral blood of the patients and their parents. Screening by Multiplex-ligation-dependent probe amplification method (MLPA) (kit SALSA P036-E1, MRC HOLLAND, Amsterdam, The Netherlands) was used for subtelomeric analysis and fluorescent *in situ *hybridization (FISH) analysis (ToTel Vysion kit, Vysis, Abbott Molecular, Illinois, U.S.A.) was subsequently used as confirmation method.

To further characterize the rearrangement extent and breakpoints an array-CGH using the Human CGH Microarray Kit 400 K (Agilent Technologies, Palo Alto, CA, USA) covering the whole genome with a 5.3 Kb overall median probe spacing was performed following the manufacturer's protocol.

### Case report

The patient is the first child of healthy, non-consanguineous parents. Karyotype was normal male. No family history of congenital anomalies or mental retardation was referred. The child was born after 36 weeks of uneventful pregnancy, by caesarean section. At birth, weight was 2.400 kg (10th-25th centile); length and head circumference were not reported. Apgar score was 9 at first minute. He showed a regular physical and psychomotor development (sitting at 6 months, walking at 14 months). At school learning difficulties were observed and a neuropsychological evaluation was performed. A borderline I.Q. level, measured with Wechsler Intelligence Scale for Children-Revised (WISC-R), associated with a deficit in graphic test of Perceptual Organization (Bender-Santucci test) and language disorders with phonological impairment, dyslexia and dyscalculia were noticed.

At the age of 8 years dropping off to sleep an episode of tonic clonic seizures at right hemi-body occurred for which he was hospitalized.

At physical examination (9 years), weight was 26 kg (50th centile), height 123 cm (10th -25th centile), head circumference 55 cm (>50th centile). In addition epichantal folds, joint hyperlaxity and three abdominal cafè-au-lait spots were noticed. Ophthalmologic evaluation showed divergent strabismus at the right eye, myopia and retinal spots without clinical significance. Cerebral MRI identified mild ectopia of cerebellar tonsilla at the foramen magnum. Abdominal ultrasound examination, cardiological examination and auditory evoked potentials were normal. Electroencephalogram showed aspecific anomalies. At the age of nine years a second episode similar to the precedent (characterized by tonic-clonic seizure at right hemi-body at dropping off to sleep) occurred causing a post-ictal paresis at right hemi-body during 10 minutes. The EEG showed centrotemporal spikes in the left hemisphere, activated by sleep and a treatment with OXC was started.

The brother was born after 36 weeks of uneventful pregnancy, by caesarean section. At birth, weight was 2.080 kg (3rd -10th centile), length 44 cm (3rd -10th centile), head circumference 31.5 cm (10th centile). Apgar score was 7 and 9 at first and fifth minute, respectively. As an infant he presented a regular psychomotor development with a delayed language for which he needed a school support.

At 4 years and 6 months the neuropsychological evaluation revealed normal non-verbal performances at psychometric test but difficulties in both expressive and comprehensive languages, with lexical and syntactic impairment. On examination (7 years), weight was 26 kg (75th centile), height 122 cm (50th centile) and head circumference 52.5 cm (>50th centile). At physical examination he presented straight eyebrows, short and smooth philtrum, right single palmar crease, shallow scrotum, one cafè-au-lait spot at the back, dry skin. Abdominal ultrasound examination, cardiological and ophthalmologic evaluation, electroencephalogram and auditory evoked potentials were normal. Laboratory investigations for metabolic disorders and molecular analysis for fragile-X syndrome were negative.

The father has completed his studies as a dentist, he doesn't present any physical impairment and has been considered healthy and normal all his life.

**Figure 1:**
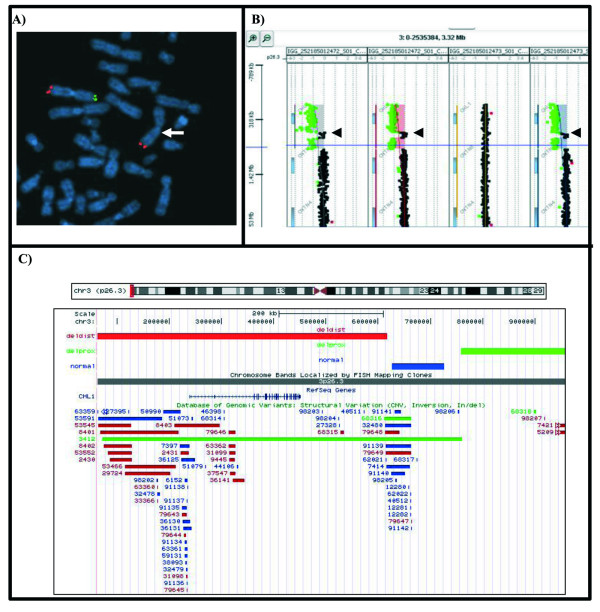
FISH and array CGH results. A) FISH with telomeric probes specific for the 3qter (red signals) and 3pter (green signal) regions. The arrow shows the absence of signal on the short arm of a chromosome 3. B) Array-CGH graphical overview of the 3p26.3 non contiguous terminal deletion. The region is deleted for the distal ~555.4 Kb from 62,075 Kb (A_18_P14035586) to 617,474 Kb (A_18_P14033572) and for the proximal ~199 kb from 758,905 Kb (A_16_P16103981) to 957,743 Kb (A_16_P16104360). Arrows indicate the non-deleted segment which spans from 626,187 Kb (A_16_P16103673) to 726,704 Kb (A_16_P36141085). From the left, the profiles refer to: first child, second child, normal mother and father. C) Custom image from UCSC Genome Browser showing an overview of the Refseq genes and CNVs content in the proximally deleted, normal and distally deleted regions.

Karyotype of the two brothers and their parents were normal. Diagnosis was done after multiplex-ligation probe amplification (MLPA) analysis of subtelomeric regions. A deletion of the probe 1721-L01329 (*CHL1*) in both sibs and their father was identified and subsequently confirmed by FISH analysis using the commercial probe TelVysion 3p (Vysis) containing the marker D3S4559 corresponding to the *CHL1 *gene and mapping on the short arm of chromosome 3 (Figure 1A). Array comparative genomic hybridization (aCGH) analysis of the three individuals showed an identical terminal deletion (Figure 1B). The 3p26.3 region was deleted for the distal ~555.4 Kb (Figure 1B), from oligomer A_18_P14035586 (62,075 Kb) (first deleted) to oligomer A_18_P14033572 (617,474 Kb) (last deleted), containing only the RefSeq gene *CHL1*. A subsequent non-deleted region was observed from oligomer A_16_P16103673 (626,187 Kb) to oligomer A_16_P36141085 (726,704 Kb) and a second deleted segment was identified for the proximal ~199 Kb from oligomer A_16_P16103981(758905 Kb) to oligomer A_16_P16104360 (957743 Kb). No Refseq genes are present within this region. Overall the three regions are only partially overlapping with numerous small putative benign copy number variations (CNVs) described in the Database of Genomic Variants (http://projects.tcag.ca/variation, build 37.1, Feb 2009). In addition they are mostly represented by duplications and no individuals with CHL1 deletions are described (Figure 1C).

Additionally, a few CNVs, spanning from 103.5 to 696 Kbs, have been identified along the genome in the two brothers and their parents (Table 1). Among them, a maternally inherited 1q44 duplication of 696 Kbs is shared by the two brothers and absent in the father.

**Table 1:** List of the additional copy number variations (CNVs) identified in the family.

Case	CNV's coordinates	Size (Kbs)	Gain/Loss	Parental origin	Presence in DGV*
**Patient**	Chr1:246,713,074-247,409,060	696	Gain	maternal	partially

	Chr2: 89,135,619-89,534,147	398.5	Loss	maternal	yes

**Brother**	Chr1:246,713,074-247,409,060	696	Gain	maternal	partially

	Chr2: 89,135,619-89,312,590	177	Loss	paternal	yes

**Father**	Chr2: 246,713,074-89,312,590	177	Loss	unknown	yes

**Mother**	Chr1:16,927,124-17,253,438	326.3	Gain	unknown	yes

	Chr1:104,107,589-104,211,056	103.5	Gain	unknown	yes

	Chr1:246,713,074-247,409,060	696	Gain	unknown	partially

	Chr2: 89,135,619-89,534,147	398.5	Loss	unknown	yes

	Chr4:160,294,949-160,578,715	283.8	Loss	unknown	no

* Database of Genomic Variants (http://projects.tcag.ca/variation, build 37.1, Feb 2009)

## Discussion

**Figure 2:**
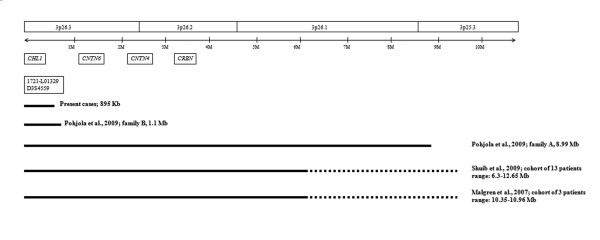
**Schematic representation of the previously reported 3p deletions analyzed by aCGH for which the extension of the deleted segment was reported**. The black lines below the chromosomal 3p 26.3p25.3 region indicate the deletions whose references and extensions are indicated on the right.

The 3p deletion syndrome is a rare contiguous gene syndrome caused by 3p25-pter deletions variable in size and mostly occurring *de novo*. The phenotype is recognizable and varies from normal to severe. Here, we describe a family with two affected children presenting a submicroscopic 3p26.3 non-contiguous terminal deletion inherited from the normal father. Overall the region spans less than 1 megabase and includes only one Refseq gene named *CHL1 *which has been previously proposed as responsible for cognitive impairment in individuals with 3p terminal deletions [8,11]. The two brothers have a mild mental retardation characterized by learning and language difficulties, but not the distinct features usually described in association with the 3p deletion syndrome. A 1,5 Mb minimal critical region including two genes, *CRBN *and *CNTN4*, has been suggested to play a causative role in the aetiology of 3p- syndrome (Figure 2). Moreover, loss of *CHL1 *was proposed to play an additional role on the cognitive impairment of the affected individuals [8]. At our knowledge, only one similar case has been reported [4]. The authors describe a terminal 3p deletion of 1.1 Mb including only the *CHL1 *locus and transmitted from the normal mother to her affected son. The index patient presents facial features different from those previously reported in the 3p deletion syndrome and skin pigmentation, an unspecific feature not related to this syndrome. Interestingly, mild learning difficulties and severe disability in language were observed. The *CHL1 *gene encodes for a member of the L1 family of neural cell adhesion molecules. These proteins play an important role in the building and functioning of the brain because a lot of events, like migration and synaptogenesis, require cell to cell and cell to matrix interactions.

*CHL1 *has been mapped to 3p26.3, and was proposed as a candidate gene for non specific mental retardation because it is highly expressed in the brain [4,8,12-14]. The finding of a balanced translocation disrupting the gene *CHL1 *in an individual affected by non specific mental retardation further supports this suggestion [11].

Our findings strongly confirm the evidence that a loss of the more proximal genes is required to cause the typical 3p- syndrome clinical features, moreover the presence of a small terminal 3p deletion including only the *CHL1 *gene can determine only a mild phenotype or no symptoms at all as we observed in the members of our family.

Since the introduction of array-CGH analysis the finding of a transmitted chromosomal variant from a phenotypically normal parent has became more frequent. Inherited CNVs associated with both abnormal and normal phenotype have been recently reviewed in 200 families[13]. Thrombocytopenia-Absent Radius syndrome [15], del(1)(q21.1)[16] and deletions and duplications of 16p13.11 region[17] represent only a few examples of genetic variations transmitted from an apparently normal parent to an affected child. A bias of ascertainment, chromosomal non-penetrance and gene modification are hypothesized as possible explanations [6]. Moreover an apparently unaffected parent who carries the deletion could also have subtle phenotypic features consistent with the deletion that would become evident on further clinical evaluation. Other possibilities may account for phenotypic variability including differences in genetic background, epigenetic phenomena, expression or regulatory variation, and the unmasking of recessive variants residing on the other allele. Disease type and severity may be explained by the occurrence of additional rare events and their inheritance within families. The combination of two large CNVs in a single individual would increase or decrease the dosage for different genes creating a sensitized genomic background [18].

The "two-hit" model, as proposed by Girirajan et al [19], wherein a secondary rearrangement event is necessary to show the phenotype, could be an alternative explanation for the differences between the father and his sons.

We documented, in fact, the two affected individuals had an additional chromosomal abnormality larger than 500 Kbp and affecting the 1q44 region (Table 1). We can speculate this maternally inherited duplication, which is neutral in the mother, could instead contribute to the differences in the disease outcome observed between the two siblings and their father.

In conclusion, this familial case, characterized by a transmitted 3p deletion containing only the *CHL1 *gene and associated with a strong phenotypic variability, confirms the hypothesis that the association of terminal 3p deletion, mental retardation and learning disabilities is not casual.

## Competing interests

The authors declare that they have no competing interests.

## Consent

Written consent was obtained from the parents of our two patients for publication of this case report. A copy of the written consent is available for review by the Editor-in-Chief of this journal.

## Authors' contributions

This paper reports the results of a multicenter study: clinical evaluation of patients has been performed by MTD, ML, MMB, MM; PR, SG, FB performed cytogenetic and molecular studies; CC and GG prepared the manuscript and participate to cytogenetic and molecular studies. All authors read and approved the final manuscript.
